# Prevalence of Corneal Astigmatism in Patients before Cataract Surgery in Western China

**DOI:** 10.1155/2020/5063789

**Published:** 2020-08-31

**Authors:** Wei Ma, Chengguo Zuo, Weirong Chen, Shaoyang Zheng, Jiangang Xu, Ruowen Gong, Maierhaba Mijiti, Kaidiliya Alifu, Lin Ding, Mingkai Lin

**Affiliations:** ^1^State Key Laboratory of Ophthalmology, Zhongshan Ophthalmic Center, Sun Yat-Sen University, Guangzhou, China; ^2^Eye Institute, Eye and ENT Hospital, College of Medicine, Fudan University, Shanghai, China; ^3^People's Hospital of Xinjiang Urumqi Autonomous Region, Urumqi, China

## Abstract

**Purpose:**

To investigate the demographics and distribution of corneal astigmatism before cataract surgery in patients from western China and to compare and analyze these findings with those of patients in southern China. *Setting*. People's Hospital of Xinjiang Uygur Autonomous Region.

**Design:**

Clinical-based cross-sectional study.

**Methods:**

Patients undergoing cataract surgery in the People's Hospital of Xinjiang Uygur Autonomous Region from February 2012 to August 2019 were recruited. Preoperative keratometric data measured by performing preoperative bilateral partial coherence interferometry (IOLMaster), and patient demographics were recorded and analyzed.

**Results:**

This study comprised 12,236 eyes of 7065 patients with a mean age of 64.75 ± 9.66 years, and 52.77% of the patients were female. The mean axial length was 23.14 ± 0.96 mm. Astigmatism ranged from 0 diopters (D) to 6.94 D, with a mean of 1.28 D. Corneal astigmatism was between 0.25 D and 1.25 D in 53.71% of eyes, 1.25 D or higher in 39.06% eyes, and less than 0.25 D in 7.23% of eyes. Astigmatism was with the rule (WTR) in 41.94% of the patients and against the rule (ATR) in 38.80% of patients. The mean flat and steep keratometry measurement was 43.19 ± 1.50 D and 44.24 ± 1.62 D, respectively. After matching, corneal astigmatism in western China was 1.30 ± 1.03 D, and it was significantly higher than that in southern China (0.98 ± 0.67 D, *P* < 0.001). After matching, the proportion of WTR astigmatism was 40.99% in western China, which was also significantly higher than the proportion (26.46%) in southern China (*P* < 0.001).

**Conclusion:**

Corneal astigmatism in patients before cataract surgery in western China was mainly between 0.25 D and 1.25 D. Compared with patients in southern China, patients in western China are younger, have a much higher degree of astigmatism, and have a higher proportion of WTR astigmatism.

## 1. Introduction

With the development of China's economy and the transition to an aging society, the need for cataract treatment to improve visual quality is increasing each year. To date, the most effective treatment is surgery. The increasing cataract surgical rate and wide application of astigmatic correction intraocular lenses provide a guarantee for this demand. Therefore, preoperative corneal astigmatism in patients has attracted extensive attention [1, 2]. Some studies have observed the distribution of astigmatism in regions such as Brazil [3], northeastern India [4, 5], southern Europe [6], and the United Kingdom [1, 7]. In China, there are only studies reported in the southern, northern, and central [8–10] regions.

However, to date, no systematic observation of corneal astigmatism in the western region of China has been seen. Considering the large geographical differences between western China and other areas of China, we assume that the astigmatism of patients is likely to be different, and it is meaningful to evaluate the distribution of western Chinese patients.

Thus, we conducted this study to investigate corneal astigmatism in patients before cataract surgery in western China.

## 2. Methods

### 2.1. Data Collection and Inclusion Criteria

This cross-sectional study recruited consecutive patients with cataracts during preoperative screening from February 2012 to August 2019 at the People's Hospital of Xinjiang Uygur Autonomous Region. This study was approved by the Human Research Ethics Committee of the People's Hospital of Xinjiang Uygur Autonomous Region and was in accordance with the tenets of the Declaration of Helsinki.

A full ophthalmologic examination of these patients included visual acuity, refraction, slit lamp evaluation, including tonometry, and dilated fundus evaluation. Exclusion criteria were patients with previous ocular surgery, including refractive surgery, history of corneal disease or intraocular inflammation, penetrating eye trauma or corneal trauma, and irregular astigmatism. Eyes with axial length (AL) greater than 26 mm or AL less than 20 mm were also excluded. All patients underwent IOLMaster 500 (Carl Zeiss Meditec, Jena, Germany) examinations by trained technicians. The flat and steep keratometry (K) and AL were recorded. The differences in corneal astigmatism between western and southern China were also compared. The distribution of corneal astigmatism in southern China was derived from our previously published data [8]. The data is comparable to the southern investigation as the same examination principle is followed.

### 2.2. Statistical Analysis

Statistical analysis was performed using SPSS for Windows version 25 software (SPSS Inc., Chicago, IL, USA). Age, axial length (AL), anterior chamber depth (ACD), corneal astigmatism (CYL), flat keratometry (K1), and steep keratometry (K2) were expressed as the mean ± standard deviation (SD) with the range. When comparisons were made between male and female groups, the independent samples *t*-test was used. When the nonnormally distributed data were compared among age groups, the Kruskal–Wallis test was used. Categorical data were evaluated via the chi-square test. Propensity matched analysis (PMS) was used to control the interference of confounding factors. A value of *P* < 0.05 was considered statistically significant.

## 3. Results

**Figure 1:**
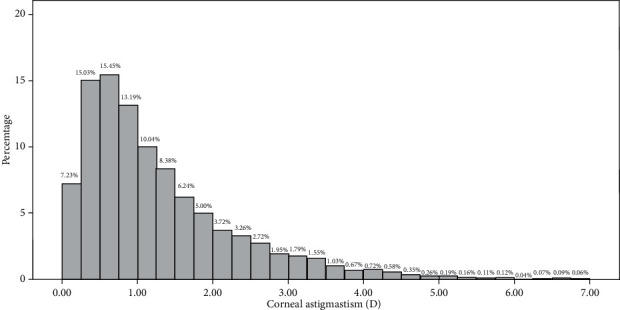
Distribution of corneal astigmatism.

A total of 12,236 eyes of 7065 patients with cataracts were enrolled in this study, including 3728 (52.77%) female and 3337 (47.23%) male patients. The demographic and clinical features of the patients are summarized in Table 1. The mean age of these patients was 64.75 ± 9.66 years, and the mean corneal astigmatism was 1.28 ± 1.04 D. According to Figure 1, the most common range of corneal astigmatism was between 0.25 D and 1.25 D in 6572 eyes (53.71%). It was higher than 1.25 D in 4779 eyes (39.06%) and less than 0.25 D in 885 eyes (7.23%).

The biometric parameters of each gender and age group are shown in Table 2. Significant differences in corneal astigmatism were observed for each gender (*P* < 0.001) but not in each age group (*P*=0.44). Increases in both K1 and K2 with age were observed (*P*=0.007 and 0.04, respectively). Corneal astigmatism was higher in male patients (1.32 ± 1.06 D) than in female patients (1.25 ± 1.03 D). On average, female patients had steeper corneas than male patients. Female patients had higher K1 values than male patients, and the K1 values of female and male patients were 43.27 ± 1.50 D and 43.10 ± 1.49 D, respectively (*P* < 0.001). The K2 values of female and male patients were 44.23 ± 1.63 D and 44.25 ± 1.60 D, respectively (*P*=0.45). The axial length was 23.14 ± 0.97 mm and 23.14 ± 0.94 mm in female patients and male patients, respectively, with no statistically significant difference (*P*=0.66).

**Figure 2:**
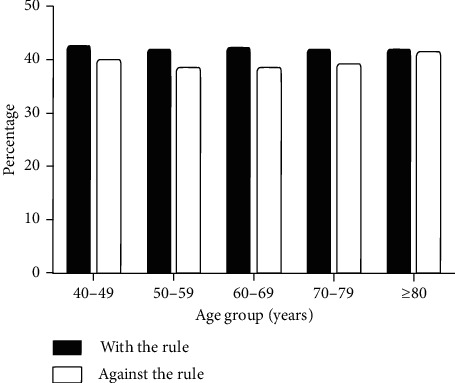
Proportion of WTR and ATR astigmatism in each age group.

The proportion of patients with astigmatism with the rule and against the rule by gender and age group has been summarized in Table 2 and Figure 2. There was no significant difference by gender or age group (*P*=0.36 and 0.77, respectively).

PMS was applied to control confounding factors, including sex and age, to compare the 2 cohorts; one is the cohort in the present study, and the other is the patients from southern China in our previous study [8]. The propensity score is shown in Table 3. The ARC-w group represents the patients in western China for this study, and ARC-s represents the patients in the southern area. After the patients were matched by the confounding factors of gender and age, significant differences could be observed between the two cohorts in CYL, K1, K2, AL, and astigmatism types (*P* < 0.001), revealing that patients in western China had significantly higher corneal astigmatism (1.30 ± 1.03 D for ARC-w and 0.98 ± 0.67 for ARC-s), flatter corneas (K1 and K2 were 42.92 ± 1.51 D and 43.97 ± 1.64 D, respectively, in ARC-w and 43.76 ± 1.52 and 44.74 ± 1.55, respectively, in ARC-s) and a longer axis (23.88 ± 0.90 mm and 23.48 ± 0.90 mm, respectively, in ARC-w and ARC-s) than did patients in southern China.

## 4. Discussion

The mean corneal astigmatism for this cohort in this study was 1.28 ± 1.04 D (range 0 to 6.94 D), which was higher than the values reported for most of the other populations, including Asian patients, European patients, and Brazilian patients, including the corneal astigmatism reported by Lekhanont et al. in patients from Thailand (1.05 ± 0.62 D), by Khan and Muhtaseb in patients from the UK (1.03 ± 0.728 D) and by Zvornicanin in patients from Bosnia and Herzegovina (0.72 ± 0.61 D) [1, 6, 11]. In one study investigated by Duman in Caucasian cataract surgery patients, 0.84 ± 0.70 D was observed [12] and another study reported the corneal astigmatism of 1.02 ± 0.69 D in Caucasian patients in an Italian hospital [13]. Corneal astigmatism in western China is also by far one of the highest corneal astigmatisms reported in the world. The reason why western Chinese patients have higher corneal astigmatism may be related to genetic and environmental factors. One study by Han and Kim investigated corneal astigmatism in patients from northern United Arab Emirates [14] and reported a similar level of corneal astigmatism of 1.32 ± 0.97 D. Similar environment of the two areas may provide a clue regarding the similarly higher levels of corneal astigmatism. Northern Xinjiang [15] and northern United Arab Emirates [16] are both located in Eurasia, with similar strong wind, sand, and ultraviolet environment. The other areas in China have lower corneal astigmatism than the western area in the present study. Yuan et al. reported 1.09 ± 0.77 D from northern China [9], and Yu et al. reported 1.15 ± 0.84 D from central China [10]. Our previous study observed 1.01 ± 0.69 D from southern China. Therefore, corneal astigmatism in the western area is higher than that in the northern, middle, and southern areas of China. After patients were matched on the confounding factors of age and gender by PMS, CYL was 1.30 ± 1.03 D and 0.98 ± 0.67 D (*P* < 0.001) for western and southern Chinese patients, respectively. The distribution of CYL is also different from that reported for other populations. A total of 39.06% of eyes had corneal astigmatism higher than 1.25 D, which is much higher than that of southern Chinese patients, in which astigmatism was higher than 1.25 D in 27% eyes. This may be due to environmental differences caused by geolocation. Xinjiang Province has higher level of ultraviolet rays and longer period of sunshine and higher altitude which contribute to oxidative stress. It has been considered to be related to corneal diseases such as keratoconus [17, 18].

The finding that the percentage of WTR astigmatism has no significant association with age is not consistent with the findings for populations from different countries and regions [2, 19]. With increasing age, the incidence of ATR astigmatism has a significant increasing trend, while WTR astigmatism tends to decrease accordingly, which can be clearly observed in southern China and in studies conducted in Europe, Brazil, Thailand, and many other areas. This finding has not been confirmed in this study (see Figure 2). After the participants were matched on the propensity scores (Table 3), the effects of age and gender were excluded. The proportion of patients with ATR astigmatism in southern China is higher than that in western China. The exact cause of the corneal curvature shift from WTR to ATR with age not clear; some studies show pressure from the eyelids, and physiological changes in the corneal structure with age may lead to this outcome [20]. According to a previous study, people in the western region have fewer epicanthi, a greater height of palpebral fissure, higher rates of double eyelid, and greater amplitude of upper eyelid movement [21]. Therefore, the eyelid pressure of people in western China has a relatively lower vertical influence and a higher horizontal influence on the cornea. These may partly explain the lower proportion of ATR astigmatism and the higher proportion of WTR astigmatism in western China. Moreover, the proportion of WTR astigmatism in the western China population is significantly higher, while the ATR astigmatism is significantly lower, and eyelid pressure which accelerates the shift from WTR to ATR in patients from western China was relatively low. Therefore, even if we observed the downward trend of WTR astigmatism with age and the trend of ATR astigmatism with age, we still could not find its statistical significance.

The mean age of the cohort in this study was 64.75 ± 9.66 years (range 40 to 103), which was younger than the mean age of the cohort in southern China (70.56 ± 9.55 years (range 40 to 95) [8]), the age figure reported by Theiss et al. in a Brazilian population (77.13 ± 9.55 years (range 49 to 92) [3]), and the figures reported by Lekhanont et al. (74.9 ± 9.8 years (range 30 to 94) in Thailand's population [11]). In terms of gender, women in western China accounted for 52.77%, while women in southern China accounted for 61.74%, which was significantly higher than the former. Therefore, different ages and gender composition ratios are also responsible for the different distribution of astigmatism in western China. AL is one of the most important indicators when calculating the required IOL power. Although the statistically significant difference was observed between AL in southern and western China, it did not have clinical significance. Mean AL in the current study is similar to that of southern China.

With the improvement of economy and medical treatment level, elderly cataract patients have higher requirements on visual quality. The use of astigmatism correcting IOLs such as toric IOLs can improve the visual quality and life quality of elderly cataract patients [22]. However, our study found that there are differences in corneal astigmatism between elderly cataract patients in western China and elderly cataract patients in southern China. Therefore, looking for the difference between the two and analyzing the reasons for the difference between them will help to select and even design astigmatism correcting IOLs in patients in western China.

To the best of our knowledge, this is the first study focused on the demographics and distribution of corneal astigmatism before cataract surgery in patients from western China. The study was a hospital-based, cross-sectional study and thus had a bias at baseline. It may not be representative of the entire population. Although challenging, these results need to be confirmed in a community-based epidemiologic survey. And we did not analyze the connection between the biometric parameters and the stages and severity of cataract, careers, and other factors.

In conclusion, this study evaluated the distribution of corneal astigmatism for cataract patients before surgery in western China due to the large differences between the environmental conditions in western China and other regions of China. The distribution of corneal astigmatism in patients before cataract surgery in western China was mainly between 0.25 D and 1.25 D, and the proportion of WTR astigmatism had no relationship with age. Compared with patients in southern China, patients in western China are younger and have much higher astigmatism and a higher proportion of WTR astigmatism.

## Figures and Tables

**Table 1:** Demographic and clinical characteristics of the ARC patients.

Characteristic	Value
Eye/patient (*n*)	12236/7065

Age (*y*)	
Mean ± SD	64.75 ± 9.66
Range	40, 103

Male/female sex (*n*)	3337/3728

Corneal astigmatism (D)	
Mean ± SD	1.28 ± 1.04
Range	0, 6.94

Flat keratometry (D)	
Mean ± SD	43.19 ± 1.50
Range	35.90, 48.98

Steep keratometry (D)	
Mean ± SD	44.24 ± 1.62
Range	36.29, 51.45

Axial length (mm)	
Mean ± SD	23.14 ± 0.96
Range	20.01, 25.99

ARC: age-related cataract; SD: standard deviation; D: diopter.

**Table 2:** Comparisons of parameters of the ARC patients by sex and age group.

Group	CYL (D) mean ± SD	K1 (D) mean ± SD	K2 (D) mean ± SD	AL (mm) mean ± SD	Astigmatism type		
					WTR (%)	ATR (%)	Oblique (%)

Sex							
Male	1.32 ± 1.06	43.10 ± 1.49	44.25 ± 1.60	23.14 ± 0.94	42.38	38.12	19.50
Female	1.25 ± 1.03	43.27 ± 1.50	44.23 ± 1.63	23.14 ± 0.97	41.55	39.40	19.05
*P* value	<0.001	<0.001	0.45	0.66		0.36	

Age							
40–49	1.25 ± 1.03	43.05 ± 1.47	44.13 ± 1.57	23.15 ± 0.99	42.30	40.19	17.51
50–59	1.27 ± 1.05	43.16 ± 1.50	44.21 ± 1.60	23.12 ± 0.97	41.86	38.37	19.77
60–69	1.29 ± 1.09	43.19 ± 1.49	44.24 ± 1.62	23.12 ± 0.93	41.94	38.37	19.69
70–79	1.30 ± 1.07	43.22 ± 1.50	44.26 ± 1.62	23.19 ± 0.97	41.54	39.24	19.22
≥80	1.23 ± 0.82	43.32 ± 1.57	44.41 ± 1.78	23.16 ± 0.96	41.45	41.45	17.10
*P* value	0.44	0.007	0.04	0.003		0.77	

ARC: age-related cataract; CYL: corneal astigmatism; K1: flat keratometry; K2: steep keratometry; AL: axial length; WTR: with the rule; ATR: against the rule; D: diopter.

**Table 3:** Demographic characteristics of the 2 ARC groups before and after matching for corneal astigmatism.

Variables	Before matching		*P* value	After matching		*P* value
	ARC-w	ARC-s		ARC-w	ARC-s	

Age (*y*)						
Mean ± SD	64.75 ± 9.71	70.56 ± 9.55	<0.001	69.21 ± 9.03	69.21 ± 9.03	>0.99
Range	40, 103	40, 95		40, 94	40, 94	
M/F (*n*)	3337/3728	1090/1661	<0.001	1022/1459	1022/1459	>0.99

CYL (D)						
Mean ± SD	1.28 ± 1.04	1.01 ± 0.68	<0.001	1.30 ± 1.03	0.98 ± 0.67	<0.001
Range	0, 6.94	0.05, 6.59		0, 6.92	0.05, 6.59	
K1 (D)	43.19 ± 1.50	43.77 ± 1.53	<0.001	42.92 ± 1.51	43.76 ± 1.52	<0.001
K2 (D)	44.24 ± 1.62	44.76 ± 1.56	<0.001	43.97 ± 1.64	44.74 ± 1.55	<0.001
AL (mm)	23.14 ± 0.95	23.45 ± 0.90	<0.001	23.88 ± 0.90	23.48 ± 0.90	<0.001

Type of astigmatism						
WTR (%)	41.80	25.08		40.99	26.46	
ATR (%)	38.89	58.26	<0.001	40.71	56.74	<0.001
Oblique (%)	19.31	16.66		18.30	16.80	

ARC-w: age-related cataract in the western area; ARC-s: age-related cataract in the southern area; M: male; F: female; CYL: corneal astigmatism; K1: flat keratometry; K2: steep keratometry; AL: axial length; WTR: with the rule; ATR: against the rule; D: diopter.

## Data Availability

The data used to support the findings of this study are available from the corresponding author upon request.
