# Corrigendum to “Prevalence of Corneal Astigmatism in Patients before Cataract Surgery in Western China”

**DOI:** 10.1155/2021/4318290

**Published:** 2021-01-16

**Authors:** Wei Ma, Chengguo Zuo, Weirong Chen, Shaoyang Zheng, Jiangang Xu, Ruowen Gong, Maierhaba Mijiti, Kaidiliya Alifu, Lin Ding, Mingkai Lin

**Affiliations:** ^1^State Key Laboratory of Ophthalmology, Zhongshan Ophthalmic Center, Sun Yat-Sen University, Guangzhou, China; ^2^Eye Institute, Eye and ENT Hospital, College of Medicine, Fudan University, Shanghai, China; ^3^People's Hospital of Xinjiang Urumqi Autonomous Region, Urumqi, China

In the article titled “Prevalence of Corneal Astigmatism in Patients before Cataract Surgery in Western China” [1], the contact details of Lin Ding were not included. The updated corresponding author information is shown above.

## References

[1] Ma W., Zuo C., Chen W. (2020). Prevalence of corneal astigmatism in patients before cataract surgery in Western China. *Journal of Ophthalmology*.

